# Supplementary material for: Engineering the Unicellular Alga Phaeodactylum tricornutum for Enhancing Carotenoid Production
Source: Antioxidants (Basel). 2020 Aug 16;9(8):757. doi: 10.3390/antiox9080757 (PMC7465010; doi:10.3390/antiox9080757)
Supplement: Supplementary file 1 [file antioxidants-09-00757-s001.pdf]

Engineering the unicellular alga *Phaeodactylum tricornutum* for enhancing carotenoid production

Francesco Manfellotto, Giulio Rocco Stella, Angela Falciatore, Christophe Brunet and Maria Immacolata Ferrante

Supplementary Materials

Supplementary Table S1. List and sequence of primers used. A, Primers used to amplify the complete coding sequence of the genes putatively involved in the carotenoid biosynthetic pathway in *P. tricornutum*. The primers present a 5′ restriction site for cloning in the vector pENTR3c. B, Primers used to screen resistant colonies by PCR. C, Primers used for qPCR amplification.

	Primer name	Primer sequence 5′-3′	Restriction enzyme	Amplification fragment size (bp)
A	VDL1_fw	ACCTTTAAAAATGCGATTCGCTTGGGTGGT	DraI	1362
	VDL1_rv	GTAGCGGCCGCAAGCGTTTCGCCTTGTATTCC	NotI	
	VDE_fw	ACCTTTAAAAATGAAGTTTCTCGGTGTTACCAGC	DraI	1314
	VDE_rv	GTACTCGAGTTTTGCTGGGAGGTTTCTCG	XhoI	
	VDR_fw	ACCTTTAAAAATGAAGCTCCACCGGAAAGG	DraI	1764
	VDR_rv	GTACTCGAGGAAGGATGAGGACTACTAGCC	XhoI	
	ZEP1_fw	ACCTTTAAAAATGAAGTTTTCTACCACGGTGTCA	DraI	1698
	ZEP1_rv	GTACTCGAGGAAAACTCTGGCGTGTATAG	XhoI	
	ZEP2_fw	TTCAGTCGACAATGGGTCTTTCGTTTCTATCATTAT	SalI	1815
	ZEP2_rv	GTACTCGAGGAGTTCTTCTTTCGTAGCTG	XhoI	
	ZEP3_fw	ACCTTTAAAAATGAAAAGATCTTGCAGTATAGTCACAATCC	DraI	1674
	ZEP3_rv	GTACTCGAGGACAAACCGGCTGCGCCACC	XhoI	
	PDS_rv	GGCGGCCCTTAAGGCGCACCCAAAACCGT	EcoRI	1875
	PDS_fw	GGCGGCCGAATTCATGAAAATTCAGGACAGAGG	EcoRI	

		CTG		
	LCY_fw	GGCGGCCCTGCAGATGGTGCAAAGGTGGAATCTC	PstI	1956
	LCY_rv	GGCGGCCCTGCAGCTGGAAACCTTCCACGATTGTT	PstI	
	PSY_fw	GGCGGCCCTTAAATGAAAGTTTCGACAAAGCTCTG	EcoRI	1518
	PSY_rv	GGCGGCCGAATTTACTTGATCCAATTGGACC	EcoRI	
	LUT_fw	GGCGGCCCTTAAGTTTCGGTACCCCACGCTT	EcoRI	1617
	LUT_rv	GGCGGCCGAATTCATGTATTCCATAACTTTCTTGTCCTTAAC	EcoRI	
	VDL2_fw	ACCTTTAAAAATGAAGCGAGCCACGAGGAA	DraI	1686
	VDL2_rv	GTAGCGGCCGCAAGTTCTTGACATCTTCTGC	NotI	
**B**	FcpB. Fw	CGCCGTAAACAGCAAATCCT		
	VDL1_int_rv	CCATGCGTTTAAGCTCTCGC		773
	VDE_int_rv	AGGAAGCGACACCAATCGTT		443
	VDR_int_rv	CGTCGATCGAAGTCTGGGAG		383
	ZEP1_int_rv	TTTCGTACTGCTTGCCGAGT		772
	ZEP2_int_rv	ATCCCACAGGTTCGTGTTCC		666
	ZEP3_int_rv	CCCCGATTAAGACGTCTCCG		848
	PDS_int_rv	GGGAATCTGGTCGAACTCG		740
**C**	VDE_qPCR_fw	ACAGCATTGGCACTAACGATT		122
	VDE_qPCR_rv	TCGTCCCGTACAGGTGTTAATG		
	VDR_qPCR_fw	GAAGCCGTTCGTCATCTTGC		84
	VDR_qPCR_rv	CGTCTTGCTCTTCGATGGGA		
	ZEP3_qPCR_fw	TCACCACATCCTCAGGGCTA		162
	ZEP3_qPCR_rv	CCAATGACAAAAGCATCTTCGAT		

Supplementary Table S2. Transformation results.

Transgene combination	Number of resistant colonies	Number of screened colonies	Number of positive colonies in the PCR screening	Strains analysed by HPLC	Strains with pigments change*
VDL1	17	17	8	5	0
VDE	29	16	10	4	0
VDR	39	10	8	5	0
ZEP1	44	15	9	3	0
ZEP2	31	26	11	3	0
ZEP 3	39	29	11	8	0
PDS	19	15	2	2	0
VDR + VDE	41	21	11	11	1**
ZEP1 + ZEP2	26	20	2	2	0
VDL1 + VDR + ZEP1	7	7	2	2	0
VDR + ZEP1 + ZEP2	2	2	2	2	0
VDR + ZEP 3 + VDE	49	49	19	19	4 (2**)
LCY	14	14	12	1	0
PSY	9	9	6	1	0
LUT	5	5	1	1	0
VDL2	25	12	6	2	0
ZEP2 + VDL1	36	20	2	0	0
LUT + PDS	6	6	0	0	0
VDR + VDE + VDL1	7	7	0	0	0
LUT + VDE	1	1	0	0	0
VDE + ZEP1 + VDL1	9	5	1	0	0
ZEP1 + ZEP2 + ZEP3	13	13	0	0	0
VDR + ZEP3 + VDL1	7	7	2	0	0
VDL1 + VDE	0	0	0	0	0
PDS + LUT	0	0	0	0	0
VDR + ZEP 1 + ZEP 3	0	0	0	0	0
VDR + VDE + ZEP 2	0	0	0	0	0

*Total pigment content increased of at least 2 fold. ** Increased pigments content respect to wild type was confirmed in different separate experiments.

Transgene combination	Strain	Fucoxanthin (pg/cell)	β-carotene (pg/cell)	Diadinoxanthin + diatoxanthin (pg/cell)	Carotenoid Sum (pg/cell)
	PT WT	0,0368	0,0038	0,0209	0,0625
VDL1	#A1	0,0202	0,0013	0,0276	0,0505
	#A2	0,0230	0,0021	0,0138	0,0406
	#A3	0,0211	0,0021	0,0132	0,0378
	#A4	0,0197	0,0021	0,0096	0,0325
	#A5	0,0127	0,0015	0,0057	0,02
VDE	#B1	0,0158	0,0022	0,0079	0,026
	#B2	0,0189	0,0021	0,0089	0,031
	#B4	0,0133	0,0010	0,0078	0,0224
VDR	#C1	0,0148	0,0014	0,0101	0,027
	#C2	0,0292	0,0023	0,0242	0,0568
	#C3	0,0319	0,0034	0,0237	0,061
	#C5	0,0643	0,0042	0,02239	0,111
ZEP1	#D1	0,0401	0,0036	0,0155	0,0592
	#D2	0,0240	0,0020	0,0060	0,0321
	#D3	0,0627	0,0015	0,0177	0,082
ZEP2	#E1	0,0702	0,0017	0,0203	0,0942
	#E2	0,0758	0,0039	0,0202	0,0999
	#E3	0,0267	0,0011	0,0094	0,0381
ZEP3	#F1	0,0611	0,0021	0,01374	0,0769
	#F2	0,0629	0,0007	0,02364	0,0873
	#F3	0,0597	0,0016	0,0192	0,0805
	#F4	0,0345	0,0038	0,0203	0,0623
	#F5	0,0128	0,0007	0,0045	0,0188
	#F6	0,0127	0,0015	0,0057	0,02
	#F7	0,0159	0,0023	0,0079	0,026
	#F8	0,0189	0,0021	0,0089	0,031
PDS	#G1	0,0445	0,0036	0,0358	0,0863
	#G2	0,0459	0,0027	0,0256	0,0755
VDR + VDE	#H1	0,0459	0,0045	0,0334	0,0853
	#H2	0,0433	0,0048	0,0332	0,0831
	#H3	0,0805	0,0077	0,0416	0,1331
	#H4	0,0507	0,0061	0,0365	0,0962
	#H5	0,0271	0,0034	0,0204	0,0517
	#H6	0,0241	0,0020	0,0060	0,0321
	#H7	0,0347	0,0011	0,0094	0,0451
	#H8	0,0188	0,0014	0,0061	0,0263
	#H9	0,0342	0,0025	0,0096	0,0462
	#H10	0,0452	0,0037	0,0132	0,0635
	#H11	0,0222	0,0017	0,0068	0,0311
ZEP1 + ZEP3	#I1	0,0362	0,0045	0,0204	0,0633
	#T1	0,0243	0,0028	0,0098	0,0421
	#T2	0,1471	0,0088	0,0715	0,2274
	#T3	0,0961	0,0091	0,0702	0,1826
	#T4	0,0791	0,0074	0,0557	0,1451
	#T5	0,0393	0,0043	0,0349	0,0801

**VDR + VDE + ZEP3**	#T6	0,0299	0,0057	0,0203	0,0573
	#T7	0,0565	0,0055	0,0285	0,0921
	#T8	0,0555	0,0059	0,0407	0,1046
	#T9	0,0412	0,0045	0,0195	0,0665
	#T10	0,0561	0,0059	0,0386	0,1024
	#T11	0,0606	0,0064	0,0429	0,1123
	#T12	0,0348	0,0042	0,0297	0,0701
	#T14	0,0589	0,0044	0,0412	0,1052
	#T15	0,0723	0,0075	0,0441	0,1267
	#T16	0,0927	0,0054	0,0594	0,161
	#T17	0,0919	0,0067	0,0585	0,1610
	#T18	0,0507	0,0062	0,0365	0,0962
	#T19	0,0368	0,0038	0,0209	0,0626
**VDL1 + VDR + ZEP1**	#L1	0,0271	0,0034	0,0204	0,0517
	#L2	0,0241	0,0020	0,0060	0,0321
**VDR + ZEP1**	#M1	0,0347	0,00107	0,0094	0,0451
	#M2	0,0222	0,00174	0,0068	0,0311
**PSY**	#O1	0,0276	0,00124	0,00201	0,031
**VDL2**	#R1	0,0220	0,0014	0,0064	0,0283
	#R2	0,0257	0,0019	0,0087	0,0367
	#R3	0,0278	0,0021	0,0098	0,0399
**LUT 1**	#P1	0,0273	0,0015	0,0100	0,0393
**LCY**	#N1	0,0288	0,0014	0,0087	0,0389
	#PT WT	0,0241	0,0020	0,0096	0,0361

Supplementary Table S3. Pigments content of the transgenic strains measured by HPLC. Measurements for the initial screening were taken only once in different experiments. The wild type has been measured in different experiment.

Supplementary Table S4. HPLC measurements for pigments in triplicates, mean and standard deviation, for strains PT WT, T1, T2 and T3.

Strain	Fucoxanthin (pg/cell)	Diadinoxanthin (pg/cell)	Diatoxanthin (pg/cell)	β-carotene (pg/cell)
PT WT	0,03280124	0,017925723	0,002274805	0,003828194
PT WT	0,030450315	0,017990974	0,002100112	0,003486644
PT WT	0,031112732	0,018284786	0,002301324	0,003576518
PT MEAN	0,031454763	0,018067161	0,002225414	0,003630452
ST.DV.	0,001212209	0,00191272	0,000109322	0,000177047
T1	0,03329243	0,019996875	0,002786961	0,005770541
T1	0,037048911	0,023949292	0,003391673	0,003525903
T1	0,038014501	0,027982292	0,003442232	0,003442322
T1 MEAN	0,036118614	0,023976153	0,003206955	0,004246255
ST.DV.	0,00249471	0,003992776	0,000364603	0,001320732
T2	0,135696309	0,056260013	0,007239501	0,007729523
T2	0,131078209	0,058541785	0,007701962	0,007739616
T2	0,139830012	0,053918507	0,006892008	0,007359852
T2 MEAN	0,135534843	0,056240102	0,007277823	0,007609664
ST.DV.	0,004378135	0,002311703	0,000406335	0,000216402
T3	0,091855673	0,059721821	0,007622308	0,008661444
T3	0,091568147	0,058518507	0,00737307	0,008429623
T3	0,092026739	0,058541785	0,007803626	0,008739258
T3 MEAN	0,091816853	0,058927371	0,007599668	0,008610108
ST.DV.	0,00231748	0,000688112	0,000286169	0,000261075
